# GWAS-identified genetic variants associated with medication-assisted treatment outcomes in patients with opioid use disorder: a systematic review and meta-analysis protocol

**DOI:** 10.1186/s13643-020-01461-z

**Published:** 2020-09-01

**Authors:** Caroul Chawar, Alannah Hillmer, Stephanie Sanger, Alessia D’Elia, Balpreet Panesar, Lucy Guan, Dave Xiaofei Xie, Nandini Bansal, Aamna Abdullah, Flavio Kapczinski, Guillaume Pare, Lehana Thabane, Zainab Samaan

**Affiliations:** 1 Neuroscience Graduate Program, McMaster University, Hamilton, ON Canada; 2 Department of Psychiatry and Behavioural Neurosciences, St. Joseph’s Healthcare Hamilton, Hamilton, ON Canada; 3 Health Sciences Library, McMaster University, Hamilton, ON Canada; 4 Health Sciences Program, McMaster University, Hamilton, ON Canada; 5 Population Health Research Institute, Hamilton, ON Canada; 6 Department of Health Research Method, Evidence, and Impact, McMaster University, Hamilton, ON Canada; 7 Father Sean O’Sullivan Research Centre, St. Joseph’s Healthcare Hamilton, Hamilton, ON Canada

**Keywords:** Genome-wide association, Medication-assisted treatment, Opioid use, Treatment response, SNP, Pharmacogenetics, Systematic review, Protocol

## Abstract

**Background:**

The burden of opioid use disorder (OUD) has been increasing in North America. Administration of medication-assisted treatments (MATs) for OUD on an individual-dose basis has been shown to affect patient responses to treatment, proving to be, on occasion, dangerous. A genetic basis has been identified for some MAT responses in a candidate gene context, but consensus has not been reached for any genome-wide significant associations. This systematic review aims to identify and assess any genetic variants associated with MAT patient outcomes at genome-wide significance.

**Methods:**

The databases searched by the authors will be: MEDLINE, Web of Science, EMBASE, CINAHL and Pre-CINAHL, GWAS Catalog, GWAS Central, and NIH Database of Genotypes and Phenotypes. A title and abstract screening, full-text screening, data extraction, and quality assessment will be completed in duplicate for each study via Covidence. Treatment outcomes of interest include continued opioid use or abstinence during treatment or at follow-up, time to relapse, treatment retention rates, opioid overdose, other substance use, comorbid psychiatric disorders, risk taking behaviors, MAT plasma concentrations, and mortality rates. Analysis methods applied, if appropriate, will include random effects meta-analysis with pooled odds ratios for all outcomes. Subgroup analyses will also be implemented, when possible.

**Discussion:**

This systematic review can hopefully inform the direction of future research, aiding in the development of a safer and more patient-centered treatment. It will be able to highlight genome-wide significant variants that are replicable and associated with MAT patient outcomes.

**Systematic review registration:**

This systematic review protocol has been registered with the International Prospective Register of Systematic Reviews (PROSPERO) (registration ID CRD42020169121).

## Background

Opioid use disorder (OUD) is characterized by the *Diagnostic and Statistical Manual of Mental Disorders, 5*^*th*^ *edition* (DSM-5) as a series of physical and psychological symptoms that promote compulsive opioid-seeking behaviors and hinder the constraint of opioid consumption [1]. The World Health Organization (WHO) reports that roughly 27 million people suffered from OUD in 2016, and about 118 thousand died due to OUD-related drug use in 2015 [2]. The continual increase of opioid-related deaths in North America has called the U.S. Department of Health and Human Services (HHS) and the Ministry of Health in Canada to declare an opioid crisis and take appropriate federal action, in 2017 and 2016, respectively [3, 4].

The most prevalent OUD treatments are a combination of pharmacological and behavioral therapies, commonly known as medication-assisted treatments (MATs) [5]. The medications act as either agonists or antagonists to endogenous opioid receptors, regulating the inhibition or stimulation of the opioid reward system [6, 7]. FDA-approved MATs include methadone, buprenorphine, buprenorphine in combination with naloxone, and naltrexone [5]. In addition to those listed, Health Canada has also recently approved the use of injectable heroin-assisted treatment for severe OUD cases [8].

The regulated administration of these MATs at an individual-based dose is essential in ensuring the effectiveness of the treatment and safety of the patients, as well as averting overdose or mortality cases [9]. Methadone dosing, for example, has been shown to be a key factor in predicting treatment outcomes. Very low doses of this agonist put patients at a higher risk of relapse [10, 11], while too high doses and the induction of methadone have been associated with a higher risk of cardiac arrhythmia and mortality, respectively [9, 12].

MAT efficacy in keeping patients from illicitly using opioids has been variable [10, 11, 13], calling into question whether a genetic basis for how patients respond to treatment exists. Several genetic studies have identified variants associated with a higher risk of developing OUD and MAT metabolism or clearance [14, 15]. However, no clear consensus has been formed regarding genes that contribute to treatment outcomes, including negative ones, in OUD patients seeking treatment. Furthermore, literature has not been systematically reviewed for genetic variants of genome-wide significance in this area, to date.

## Objectives

This systematic review aims to assess all the identified genetic variants from genome-wide association studies (GWASs) significantly associated with treatment outcomes for OUD patients receiving MAT.

The specific objectives of this study include:

1. Summarizing the genome-wide significant variants associated with MAT outcomes within the current literature.
2. Comparing and meta-analyzing significant GWAS findings relevant to treatment outcomes, applying sub-group analyses based on ethnicity, sex and other variables, if possible.
3. Critically reviewing the literature to identify gaps that need to be addressed within the pharmacogenomics of MAT research.

## Methods

This protocol has been reported in accordance with the Preferred Reporting Items for Systematic Reviews and Meta-Analyses Protocols (PRISMA-P) reporting guidelines [16]. An accompanying checklist could be found in Additional file 1.

### Eligibility criteria

Studies included in this review will be limited to GWASs. Other types of genetic studies, such as candidate-gene, twin, linkage-analysis, segregation-analysis, and familial aggregation, will not be included. Studies included will also investigate a MAT in an OUD population. For the purposes of this review, study populations with opioid/heroin/fentanyl dependence, use, abuse, or addiction will be included. Examples of MATs included are methadone, suboxone, buprenorphine, naltrexone, naloxone, heroin-assisted, levacetylmethadol, and fentanyl. Studies whose participants are solely on clonidine, lofexidine, or any other opioid withdrawal medication not administered with a MAT will be excluded as these measures are for short-term management of acute withdrawal and not maintenance treatments. The inclusion of studies will not be restricted based on MAT treatment administration setting, such as community, residential, or institutional, or population characteristics, such as age, ethnicity, sex, or gender.

### Information sources and search strategy

A librarian from the Health Sciences Library at McMaster University with expertise in systematic reviews will be consulted in developing the search strategy. A unique and predetermined search strategy will be developed for exporting publications from each of the select databases and GWAS data-sharing sites. These include MEDLINE, Web of Science (All Databases), EMBASE, CINAHL and Pre-CINAHL, GWAS Catalog, GWAS Central, and NIH Database of Genotypes and Phenotypes. Studies will not be restricted by language or date of publication but will be limited to human participants if limiting by species is made possible through the database. Databases will be searched from inception until present. All sources of literature, including gray literature, will be searched. Handsearching techniques will also be applied to identify articles of interest that are not detected by the databases systematically searched. A detailed search strategy is presented in Table 1. The start date of the study is March 1, 2020.

**Table 1:** Search strategy

Medline (Ovid): 1. Genome-Wide Association Study/ 2. Genotyping Techniques/ 3. Genome, Human/ 4. Genetic Variation/ 5. genetics/or exp human genetics/ 6. (human* adj2 (genotyp* or genome* or genetic*)).ti,ab,kw,kf. 7. (GWS or GWAS or GWA).mp. 8. genome wide.ti,ab,kw,kf. 9. 1 or 2 or 3 or 4 or 5 or 6 or 7 or 8 10. exp Opioid-Related Disorders/ 11. ((opiate* or opioid* or heroin* or codeine* or dilaudid* or fentanyl* or narcotic* or drug* or substance*) adj2 (overdose* or use* or using or misuse* or abus* or dependence* or addict*)).ti,ab,kw,kf. 12. Opiate Substitution Treatment/ 13. ((opiate* or opioid*) adj2 (treatment* or therap*)).ti,ab,kw,kf. 14. exp buprenorphine/ or exp naloxone/ 15. exp Methadone/ 16. (suboxone or methadone or buprenorphine or naloxone).ti,ab,kw,kf. 17. 10 or 11 or 12 or 13 or 14 or 15 or 16 18. 9 and 17 19. *Limit 18 to humans* Web of Science—All databases: 1. TS = (genome-wide association study or genome-wide association or GWAS or GWA or genome wide or genome) 2. TS = ((opiate* or opioid* or heroin* or fentanyl* or narcotic* or drug* or substance*) NEAR/2 (overdose* or use* or using or misus* or abus* or dependence* or addict*)) 3. TS = ((treatment* or therap*) NEAR/2 (opiate* or opioid* or heroin* or fentanyl* or narcotic* or drug* or substance*)) 4. TS = (methadone or buprenorphine or naloxone or naltrexone or heroin-assisted or suboxone) 5. #3 or #4 6. #1 and #2 and #5 EMBASE (Ovid): 1. Genome-Wide Association Study/ 2. Genotyping Techniques/ 3. Genome, Human/ 4. Genetic Variation/ 5. genetics/or exp human genetics/ 6. (human* adj2 (genotyp* or genome* or genetic*)).ti,ab,kw. 7. (GWS or GWAS or GWA).mp. 8. genome wide.ti,ab,kw. 9. 1 or 2 or 3 or 4 or 5 or 6 or 7 or 8 10. exp Opioid-Related Disorders/ 11. ((opiate* or opioid* or heroin* or codeine* or dilaudid* or fentanyl* or narcotic* or drug* or substance*) adj2 (overdose* or use* or using or misuse* or abus* or dependence* or addict*)).ti,ab,kw. 12. Opiate Substitution Treatment/ 13. ((opiate* or opioid*) adj2 (treatment* or therap*)).ti,ab,kw. 14. exp buprenorphine/or exp naloxone/ 15. exp Methadone/ 16. (suboxone or methadone or buprenorphine or naloxone).ti,ab,kw. 17. 10 or 11 or 12 or 13 or 14 or 15 or 16 18. 9 and 17 19. *Limit 18 to human* CINAHL and Pre-CINAHL: 1. genome-wide association study or genome-wide association or GWAS or GWA or genome wide or genome 2. opiate* or opioid* or heroin* or fentanyl* or narcotic* or drug* or substance* 3. overdose* or use* or using or misus* or abus* or dependence* or addict* 4. S2 and S3 5. treatment* or therap* 6. S5 and S2 7. methadone or buprenorphine or naloxone or naltrexone or heroin-assisted or suboxone 8. S6 or S7 9. S1 and S4 and S8 10. *Limit to Human* GWAS Catalog—publications: - methadone - opioid - heroin - drug abuse GWAS Central—studies list: - methadone - heroin - opioid - opiate - addiction - drug abuse - opioid dependence - opioid addiction - fentanyl NIH Database of Genotypes and Phenotypes: - Search (opioid) - Search (heroin)	

## Study records

### Data management

All studies will be exported from the previously mentioned databases using the search strategy in Table 1 and imported into Zotero [17], a citation management software, where they will be screened for duplicates. We will then import studies into Covidence [18], for another round of duplicate screening and removal, title and abstract screening, full text screening, and data extraction. Each study will be screened and reviewed in duplicate through a team of 8 reviewers. In the case of any disagreements, the conflict will be resolved by a senior reviewer (CC or AH). As per the Preferred Reporting Items for Systematic Reviews and Meta-Analyses (PRISMA) guidelines [19], a flow chart detailing the stepwise screening process will be provided.

### Selection process

Studies will be screened twice in pairs; once assessing the title and abstract, and another time at the full text phase. All articles will be screened for the same inclusion criteria previously mentioned, during both screening processes. All reviewers will partake in a calibration phase to ensure that the purpose of this review and the inclusion criteria are understood by all, and that no discrepancies exist across the reviewers. Since the screening of studies will occur via Covidence, reviewers are blinded to their colleagues’ votes until after they have inputted their own votes, reducing the potential for bias.

### Data collection process

Data extraction will be completed in pairs for any articles that pass the screening process. A full text extraction form will be constructed on excel and then uploaded onto Covidence. The data extraction form will be pilot tested independently in duplicate to ensure its feasibility in this systematic review. For any missing data from studies during the data extraction phase, contact will be made with the study authors to supplement the missing data. All records of communication and contact with the authors will be documented.

## Data items

Information collected on this form will include: author(s), year of publication, country, cohort population, number of participants (separated by MAT), ethnicity of participants, mean age, sex ratio, type and dose of MAT, MAT outcomes (as outlined under “Outcome Measures”), any genetic variants found to be significantly associated with the outcomes, method of statistical measures, and *p* values. The traditional genome-wide significance threshold reported in the literature is *p* ≤ 5 × 10^−8^. However, since a considerable number of studies with a borderline genome-wide significance have been shown to be replicable and showcase genuine associations, *p* ≤ 1 × 10^−7^ will be used as the significance threshold for this review [20].

## Outcomes and prioritization

The main focus of this systematic review will be to assess GWAS-identified genetic variants significantly associated with MAT outcomes.

The primary MAT outcome of interest is illicit (unprescribed) opioid use throughout the duration of the MAT and at follow-up periods, the duration of which are to be determined based on the different studies reviewed. Continued illicit opioid use and abstinence from opioids will be assessed from urine toxicological screens and/or self-reported data.

Secondary outcomes of MAT to be considered in this review are:

1. Time to relapse, defined as the duration to the first use of illicit opioids after achieving abstinence.
2. Treatment retention, defined as the length of time a participant remains on MAT, and reasons for stopping MAT or dropping out.
3. Opioid overdose incidence, measured by self-report, adjudication of medical records, emergency admissions, opioid-related hospitalization, or use of naloxone.
4. Non-opioid substance use, self-reported or identified through urine toxicology screens.
5. Comorbid psychiatric disorders, self-reported or diagnosed.
6. Risk-taking behaviors related to drug use (i.e., injection, needle sharing), criminal activities, and social adversities, as reported in the original studies.
7. MAT and metabolite plasma concentrations and clearance, obtained through blood plasma analysis.
8. MAT doses, measured throughout the administration of MAT and at follow-up periods, as reported in the original studies
9. All-cause mortality, including opioid-related mortality.

## Risk of bias in individual studies

Quality assessment and risk of bias scores of included studies will be provided independently by each reviewer. The Quality of Genetic Association Studies (Q-Genie) tool [Version 1.1] developed by McMaster University will be used to assess both the qualitative and quantitative aspects of each study [21]. It is tailored to assess the validity and reliability of genetic association studies. Through Q-Genie, a quality score that corresponds to “low”, “moderate”, or “high” quality would be calculated for each study. The Grading of Recommendations Assessment, Development, and Evaluation (GRADE) tool will be used to assess the risk of bias, strength of evidence, and consistency of included studies [22]. Disagreements occurring between two reviewers regarding the risk of bias score will be resolved through discussion. If a unanimous decision is not reached, then, a third senior author will be consulted.

## Data synthesis

If appropriate, quantitative methods of synthesis will be applied. Heterogeneity between the studies will be assessed through the *I*^2^ statistic and 95% confidence interval. If low heterogeneity levels are observed, quantitative methods of synthesis applied will include a random effects metaanalysis with pooled odds ratios for main and secondary outcomes previously mentioned. If a large number of studies are identified in this systematic review, subgroup analyses will be used, where the studies will be separated based on the ethnicities of their respective populations and analyzed accordingly, as genetic associations might be more predominant in certain ethnic groups than others. Other subgroup analyses to be considered are based on variables observed to influence MAT outcomes. These include sex, type of MAT, type of illicit opioid used (for example, heroin versus prescription opioids), and alcohol use comorbidity, if discussed in the original studies. All statistical analysis will be conducted via the RStudio [1.1.456] interface of R statistical software [23].

## Metabias

To address the potential publication bias that might be encountered, PROSPERO and ClinicalTrials.gov databases will be searched for relevant clinical trial protocols that might not have been followed by a publication of results [24, 25].

## Confidence in cumulative evidence

To assess the risk of bias within and across studies in the systematic review proposed, GRADE will be used [22]. It will be implemented to evaluate the study limitations and biases that contribute to each outcome of interest reported. The GRADE approach will assess the effect of the limitations on the results, effects being “not serious”, “serious”, or “very serious”. Downgrading of the quality of the study will take place depending on the assessed effect level.

## Presenting and reporting of results

Results will be reported according to PRISMA guidelines, with special considerations to Human Genome Epidemiology Network (HuGENet) guidelines when applicable to GWAS data presentation [19, 26]. Though HuGENet guidelines are more pertinent to systematic reviews and meta-analyses of candidate gene studies with foci on single or multiple related genes, they will be used to uphold a standard when presenting genetic association data, when feasible. Tables will be used to present information on each genetic variant-phenotype association reported, including the study details, population, findings, and source of data. Forest plots will be used to display meta-analysis results, should a meta-analysis be appropriate to conduct. The overall quality of each published result will be discussed, taking into account the risk of bias scores.

## Discussion

This systematic review will be able to identify GWASs that have been conducted regarding MATs for OUD. Having a clear list of relevant studies will enable easier access to published results by the public and researchers alike. Results of the meta-analysis will be informative in determining if any genetic markers have been identified to have an impact on MAT outcomes in patients. This will help direct which genes are of interest for future candidate gene studies or GWASs. It will also allow for a consensus to be made regarding whether genetics affect treatment outcomes in the OUD population. Furthermore, if performed, stratified meta-analyses based on population ethnicities will contribute to the breadth of knowledge of genetic differences between ethnic groups. In addition, this review will allow for more informed treatment plans for individuals with differing ethnicities and genetic makeup. A potential limitation that could arise would be the inability to conduct subgroup meta-analyses due to high calculated heterogeneity between studies or small study numbers. In that case, the studies will be qualitatively reviewed and critically assessed according to their risk of bias scores. Another limitation of the proposed review is the exclusion of results obtained from candidate gene studies. Although some relevant SNP-outcome associations will not be reported on, the level of those reported will be of genome-wide significance, highlighting associations that can be expected and replicated in GWASs.

## Supplementary information

**Additional file 1.** PRISMA-P 2015 Checklist. This file is submitted in .pdf format and shows adherence to the PRISMA-P guidelines.

## Data Availability

Not applicable. No data were generated, analyzed, or reported in this manuscript.

## Abbreviations

OUD: Opioid use disorder
MAT: Medication-assisted treatment
PROSPERO: International Prospective Register of Systematic Reviews
DSM-5: Diagnostic and Statistical Manual of Mental Disorders, 5th edition
WHO: World Health Organization
HHS: U.S. Department of Health and Human Services
GWAS: Genome-wide association study
PRISMA-P: Preferred Reporting Items for Systematic Reviews and Meta-Analyses Protocols
PRISMA: Preferred Reporting Items for Systematic Reviews and Meta-Analyses
Q-Genie: Quality of Genetic Association Studies
GRADE: Grading of Recommendations Assessment, Development, and Evaluation
HuGENet: Human Genome Epidemiology Network
